# Attachment and self-regulation in the workplace—a theoretical integration

**DOI:** 10.3389/fpsyg.2024.1387548

**Published:** 2024-11-21

**Authors:** Queyu Ren, Anna Topakas, Malcolm Patterson

**Affiliations:** ^1^School of Business Administration, Southwestern University of Finance and Economics, Chengdu, China; ^2^Institute of Work Psychology, The University of Sheffield, Sheffield, United Kingdom

**Keywords:** attachment styles, self-regulation, work outcomes, attachment styles in the workplace, attachment orientations

## Abstract

Interest in adopting attachment theory to interpret workplace dynamics is growing, reflected in increasing theoretical development and empirical research. However, the advancement of the field has been hindered by the limited attention paid to the cognitive, affective and behavioral processes involved in carrying the effect of attachment styles on outcomes. Adopting a self-regulatory lens, this paper aims to unpack the attachment black box by integrating attachment theory and self-regulation theory. We propose a theoretical framework that explicates how attachment styles function to shape individuals’ regulatory responses from cognitive, affective, and behavioral perspectives, as well as identifying boundary conditions of the activation processes of attachment styles in the workplace. The framework provides novel insights into the effects, mechanisms, and boundary conditions of employee attachment styles in the workplace. Implications of the framework and future research directions are discussed.

## Introduction

Attachment theory, as proposed by Bowlby (1969), explains individuals’ approach to seeking closeness, shaped by past relationship experiences, resulting in trait-like dispositions known as attachment styles or orientations. Applied in workplace literature, it encompasses topics like leadership (Mayseless, 2010), employee behaviors (Geller and Bamberger, 2009), and job satisfaction (Kafetsios and Gruda, 2018). Evidence shows attachment styles reliably predict work outcomes such as workplace relationships and employee well-being (Harms, 2011; Yip et al., 2018; Scandura and Meuser, 2022), outlining how variations in internal working models influence thoughts, feelings, behaviors, and work processes (Cassidy and Shaver, 2008). However, extant literature frequently overlooks the processes through which attachment styles affect workplace outcomes (Harms et al., 2016), resulting in inconsistent findings about how these styles function in influencing outcomes such as leader-member relationships (Fein et al., 2020; Warnock et al., 2024). This “attachment black box” restricts the field’s development by leaving the mechanisms and conditions under which attachment styles affect work outcomes unclear. Such gaps not only challenge the practical application of attachment theory but also limit future research efforts, highlighting the need for deeper exploration of these processes to better understand attachment theory’s relevance to the workplace.

In light of this, we propose to unpack the “attachment black box” using a self-regulation perspective to address the gap in understanding how attachment styles influence work outcomes. Self-regulation theory suggests that managing impulses relies on finite internal resources, which, when depleted, impair one’s ability to resist automatic responses (Baumeister et al., 1998; Schmeichel and Baumeister, 2004). We argue that attachment styles shape these self-regulatory capabilities, affecting how employees interact, manage impulses, and respond to workplace dynamics. This paper integrates attachment and self-regulation theories to explain how regulatory processes—cognitive, emotional, and behavioral—mediate the impact of attachment styles on work outcomes. We suggest that early attachment experiences shape lifelong patterns (Mikulincer and Shaver, 2007), influencing perceptions of self and others in professional settings. Examining these patterns reveals the psychological mechanisms driving workplace dynamics and explains why employees with different attachment styles respond differently to similar stressors (Ogunfowora et al., 2021). This perspective extends attachment theory’s application to organizational research, offering a nuanced understanding of how individual traits and situational factors shape workplace behavior. Build on these insights, we seek to address the following research questions: How do attachment styles influence work outcomes? What are the boundary conditions for the activation of attachment styles in organizational settings?

This paper contributes to the literature in several ways. First, our paper uncovers how attachment styles function in the workplace by employing a regulatory lens to delineate the mechanisms through which attachment styles influence various outcomes. This is achieved by focusing on cognitive, emotional, and behavioral regulation. Our framework offers a nuanced interpretation of how different attachment styles vary in their perceptions of workplace dynamics, their ability to construct and maintain workplace relationships, their methods of emotional regulation within the workplace, and their behavioral orientations toward work tasks and social interactions. This comprehensive approach not only aligns with existing developmental psychology research but also extends it by contextualizing attachment theory within the unique environment of the workplace. This extension provides new insights into how attachment styles can be leveraged to enhance organizational outcomes.

Second, in line with trait activation theory, this paper highlights the contextual factors that may act as stimuli that activate individuals’ attachment styles and exert moderating effects on the regulatory processes. By categorizing contextual factors on the task level, social level and organizational level, this paper sheds light on how different attachment-relevant situations activate and augment the attachment styles in the workplace and how these situational cues interact with different attachment styles to influence work outcomes.

Last, we advance the literature on attachment styles in the workplace by offering a theoretical framework that brings together attachment theory and regulatory processes, accounting for contextual factors. By doing so, we open the black box in the relationship between attachment styles and work-related outcomes. Our objective is to unpick the potential mechanisms behind attachment styles to better understand how and why it influences and informs a variety of workplace dynamics and work outcomes.

## Foundations of attachment theory

Attachment theory, developed by Bowlby (1969), posits that early caregiver interactions shape enduring patterns of attachment behavior throughout life. Central to this theory are two dimensions of attachment insecurity: anxiety and avoidance. Attachment anxiety reflects the degree to which individuals hyperactivate their need for proximity and reassurance from others, often driven by concerns over being valued or abandoned. In contrast, attachment avoidance pertains to discomfort with closeness, leading individuals to prefer self-reliance and emotional distance, often rooted in experiences where attachment figures were unavailable or inconsistent (Brennan et al., 1998; Mikulincer and Shaver, 2007). When individuals score low on both dimensions, they exhibit secure attachment, characterized by a balanced view of themselves and others, and comfort with intimacy.

While early attachment theory focused primarily on parental relationships, its principles extend beyond childhood, encompassing various adult contexts such as romantic relationships, friendships, and workplace interactions (Fraley, 2019; Bowlby, 1973). The theory suggests that attachment styles are not static but can be activated by ‘strange situations’ or challenging circumstances at any life stage. In this paper, we employ the two-dimensional framework of attachment, widely used in organizational behavior research, to explore attachment’s relevance in workplace dynamics. Table 1 provides definitions and clarifications for the terms used throughout this discussion.

**Table 1:** Clarification of attachment-related concepts.

Attachment Theory	This is the overarching framework developed by Bowlby, which posits that early interactions with caregivers form the basis of later relational patterns and behaviors.
Attachment Style	Refers to the characteristic ways individuals think, feel, and behave in relationships, typically categorized into secure, anxious, and avoidant styles.
Attachment Orientation	Another term often used interchangeably with attachment style, focusing on the general tendency toward secure, anxious, or avoidant relational patterns.
Attachment System	It is an internalized pattern that influences how individuals seek comfort and regulate distress, which enables individuals to conserve brain resources by depending on others for various survival and self-regulatory needs.
Attachment Insecurity	Refers to the broad category of non-secure attachment styles, including both anxious and avoidant styles.

## Regulatory processes

**Figure 1:**
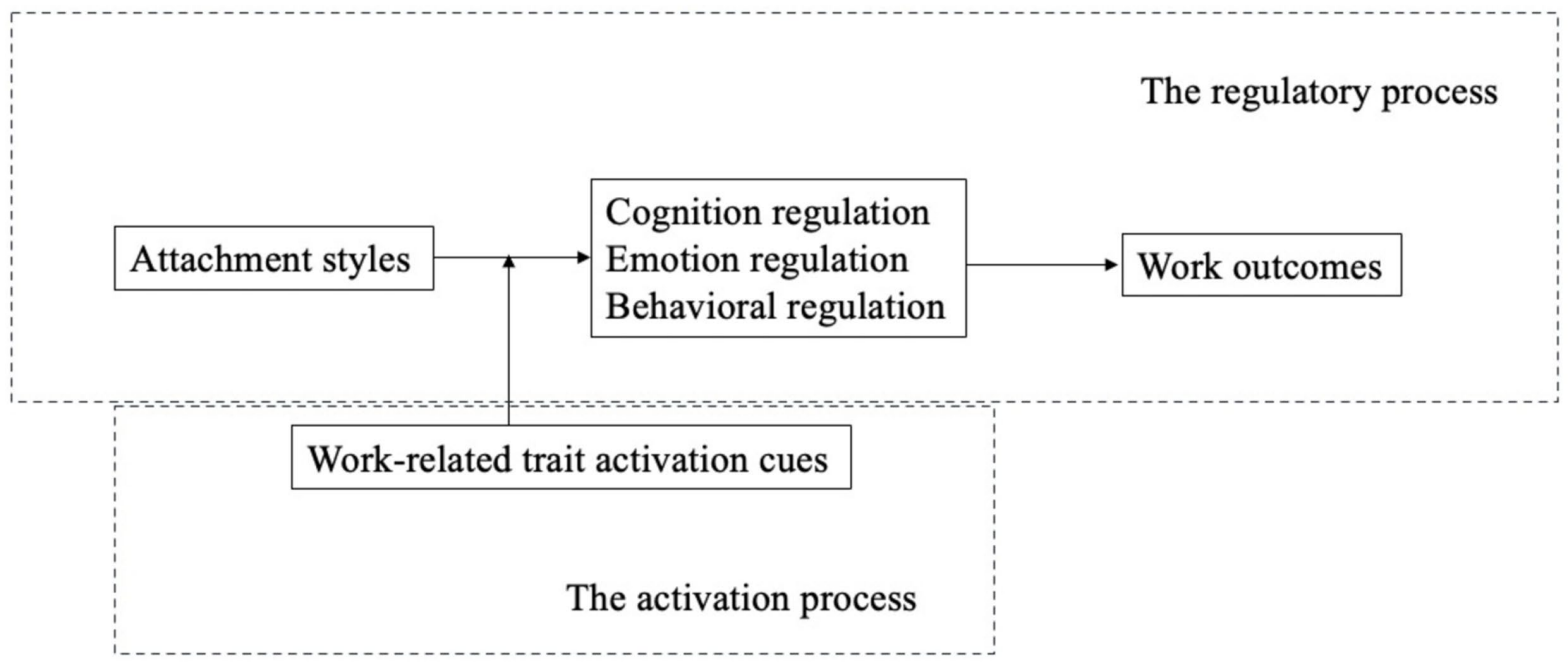
Activation and regulation processes in the relationship between attachment styles and work outcomes. This model illustrates how attachment styles (X) influence work outcomes (Y) through three self-regulatory processes: cognitive regulation, emotion regulation, and behavioral regulation (M). Work-related trait activation cues serve as a moderator, impacting the strength or direction of these self-regulatory pathways. Under certain trait activation cues, such as task demands or social context cues, employees’ attachment styles are likely to trigger specific self-regulatory responses that shape work outcomes, demonstrating how individual attachment influences workplace behavior and effectiveness.

The interplay between self-regulation theory and attachment theory provides a nuanced understanding of how limited self-regulatory resources shape attachment-driven behaviors in the workplace. Attachment theory suggests that individuals develop internal working models—mental frameworks based on early relational experiences—that guide their responses to social dynamics. Self-regulation theory complements this by highlighting that the cognitive, emotional, and behavioral resources needed to manage these responses are finite. For instance, individuals with insecure attachment styles often engage in self-regulation to manage anxieties or preoccupations in their professional interactions. However, as these resources deplete, the ability to maintain focus or constructively engage with colleagues may diminish, heightening stress or leading to avoidance of interaction. Over time, recurring depletion of self-regulatory resources due to attachment-related anxieties can impact workplace behaviors, such as decision-making or adaptability. In this way, attachment-driven responses are strongly influenced by available self-regulation resources, with workplace stressors that further tax these resources exacerbating attachment-based challenges in maintaining professional engagement and resilience. In this section, we propose a theoretical framework (Figure 1) that depicts how attachment styles influence work outcomes through self-regulatory mediating mechanisms and the activation process of the attachment styles. We start by unpicking the potential mechanisms in the following section. Specifically, we explore the mechanisms of cognitive regulation, emotion regulation and behavioral regulation.

### Attachment and cognition regulation

Cognitive regulation encompasses the management and adjustment of cognitive functions such as attention, memory, problem-solving, and decision-making. It involves employing diverse strategies to achieve goals and adapt to different situations effectively (Baumeister and Vohs, 2007; Vohs and Baumeister, 2016). Our perceptions and interpretations of the social surroundings are typically interpreted in relation to others. Consciously or unconsciously, we make subjective inferences about others and events that happen around us. The interpersonal adaptation of the human mind allows us to reflect upon our own thoughts, feelings and behaviors as well as interpret others’ manifested emotions and behaviors (Baldwin, 1992). For years, developmentalists have been exploring how people come to an awareness and interpretation of social surroundings. Fonagy et al. (1991) put forward the notion of the ‘pre-reflective’ and ‘reflective’ self, in which a pre-reflective self refers to the immediate experience of life whereas the reflective self involves the internalization and interpretation of what we experience in life. Such a ‘mentalization’ process involves reflective understanding of self and others (Fonagy et al., 2007), which directs individuals’ sensemaking and helps us navigate ambiguous social information. Through mentalization, an internal reality is formed in which we are able to interpret the mental states of others and attribute causes to their emotions or actions (Green-Hennessy and Reis, 2005). The mental realities we form are intrinsically subjective, which explains why individuals hold different perceptions of external events.

Attachment theory in essence depicts individuals’ cognitive differences in terms of processing and perceiving attachment-related information (Mikulincer and Shaver, 2007). The formation of individuals’ attachment styles is a result of close relationship history (Bowlby, 1973). In line with the notion of reflective self, the sense-making process of individuals’ attachment styles consists of an internal working model of self, which is individuals’ impression or perception of themselves in a close relationship. It also involves individuals’ reflection of others. Individuals accumulate knowledge and information about their relationships with significant others since the early stages of their development, which gradually forms a pattern (Bartholomew and Horowitz, 1991; Fraley, 2002). This pattern of attachment impressions will function as a reservoir of attachment-related information, which eventually influences their perception of current or future close relationships (Mikulincer and Shaver, 2007). When individuals are exposed to a persistent pattern of attachment behavior at an early age, these experiences become ingrained, forming mental schemas that shape their perceptions of self in relation to others (Dykas and Cassidy, 2011). These mental frameworks lead to the development of expectations regarding present and future attachment experiences. For example, we posit that if individuals are habitually involved in a consistent and caring attachment relationship, which is when their significant others have a consistent and caring manner when interacting with them, individuals will store this positive attachment information and generalize it to predict future close relationships. However, when their significant others treat them in an inconsistent way, they will be confused by the uncertainty of the relationship, causing them to doubt whether they deserve care and attention. When individuals are persistently exposed to the situation where attachment figures are unavailable or neglect their attachment needs, they will gradually form the perception that significant others are unreliable, thus becoming habitually self-reliant and distant.

In organizational settings, the perception and processing of social cues are closely linked to how individuals interpret and attribute causality to workplace interactions. Secure attachment patterns, shaped by positive past experiences, often lead to a positively biased perception of social events, which in turn fosters confidence in their self-worth and expectations of fair treatment from colleagues and supervisors (Bowlby, 1969). In contrast, attachment insecurity in the workplace points to maladaptive patterns of social cognition, where perceptions are often negatively biased or distorted (Dykas and Cassidy, 2011). A hypothetical scenario in a team setting would be: In a team setting working on a tight deadline, attachment styles can shape decision-making through cognitive regulation. A securely attached employee, feeling confident and open to input, objectively weighs both short-and long-term options, fostering balanced discussion. In contrast, an anxiously attached employee, driven by a need for social acceptance, may lean toward the majority’s preference to avoid conflict, potentially overlooking valuable insights. Meanwhile, an avoidantly attached employee, valuing independence, may prefer a solution that minimizes reliance on the team, disregarding collaborative benefits. These differences highlight how attachment styles impact cognitive regulation and influence team decisions. Another example is that employees with attachment anxiety may attribute a supervisor’s constructive criticism to job insecurity or personal inadequacy, perceiving such interactions as threats to their standing within the organization. Similarly, avoidantly attached employees may react to positive feedback or team collaboration with skepticism, doubting the authenticity or intentions behind such interactions. This pattern mirrors findings in non-work domains, where insecure individuals tend to make negative attributions in significant relationships (e.g., Gallo and Smith, 2001; Collins et al., 2006), suggesting that similar cognitive biases may manifest in professional contexts. For instance, an anxiously attached employee may perceive neutral feedback as a signal of disapproval, while an avoidantly attached employee may respond to supportive gestures with distrust, impacting their willingness to engage in collaborative tasks. These tendencies highlight the need for more workplace-specific insights into attachment styles, illustrating how attachment insecurity might influence interactions and interpretations of work-related social cues.

We perceive attachment styles not only as a cognitive process that is formulated when individuals try to make sense of social surroundings, but also as an inner resource and regulatory device (Mikulincer and Shaver, 2007). Attachment theory shares similar postulation with sociometer theory (Leary and Baumeister, 2000; Leary and Downs, 1995); they both underscore the importance of individuals’ need for belongingness. Sociometer theory focuses on self-esteem as an indicator of individual self-evaluation. Self-doubt, as a result of the inability to sustain close interpersonal relationships, is likely to activate individuals’ need for belongingness. Murray et al. (2003) found that in romantic relationships, the chronic feeling of being valued is related to higher self-esteem and positive evaluation of one’s relationship partner, which then results in more positive and proactive behaviors when facing stressful situations such as feeling hurt by their partner. This is consistent with attachment security, which helps individuals to sustain their self-esteem and positive evaluation of self and significant others.

We use the evidence and theorizing from the literature on parental and romantic relationships to inform the workplace literature and understand how attachment styles influence workplace outcomes through information processing. We detail the application of individual differences regarding individual’s social cognitive regulation strategies as twofold. First, it is easier for us to understand employees’ perception of self. The ability to receive and process social information in a relatively less biased and distortive manner is essential for individuals to develop confidence and self-esteem (Thompson and Raikes, 2003). Attachment insecurities represent an imbalance between independence and interdependence (Bretherton, 1992). For secure individuals, they are able to juggle between their need for autonomy and interpersonal relationships at work. However, anxious individuals are more socially sensitive as they worry about being neglected or rejected. Research suggests that social hypersensitivity is related to lower self-esteem (Yang and Girgus, 2018). The hyperactivation of their need for proximity is thus related to lack of confidence, meaning that anxiously attached individuals are more likely to rely on others for recognition and support. In the workplace, this is represented by employees’ constant underestimation of their capabilities and seeking for reassurance from colleagues or their leaders (Keller, 2003). Whereas avoidant individuals are shown to deactivate their need for dependence. This is usually represented by distancing themselves and unwillingness to seek closeness to others. Compared with insecure individuals, secure individuals are found to be more suited to leadership roles (Mayseless, 2010), more confident about their effectiveness as a leader and are more likely to be perceived as team leaders by teammates (Berson et al., 2006). Second, it informs our understanding of employees’ perception of others at work. Individuals bring pre-existing knowledge or perceptions to the workplace, when insecure employees interact with colleagues or their leaders, it is likely that they tend to show lack of trust toward their leaders (Frazier et al., 2015; Simmons et al., 2009; Harms et al., 2016). Anxiously attached employees lack of trust toward their leaders as they doubt their self-worth and constantly worry of being abandoned by others while avoidantly attached employees’ lack of trust originates from their doubt toward others (Harms et al., 2016).

Taken together, attachment styles signify individual differences concerning social information processing. The social construal process influences individuals’ perceptions of self and others. In work scenarios, attachment styles are able to influence work outcomes through information processing.

*Proposition 1. Employee attachment styles influence work outcomes through regulating their cognitions toward workplace dynamics*.

### Attachment and emotion regulation

Emotion regulation (ER) is a purposeful endeavor focused on shaping the intensity, duration, and nature of emotions felt or displayed (Gross, 1998). The mediating role of emotion regulation between attachment styles and work outcomes is currently under-explored, but we postulate that it serves as an important explanatory factor of how individual differences regarding attachment styles would result in different effects of employee well-being, attitude and behaviors.

Individuals adopt different emotion regulation strategies when encountering negative events. The selection of coping strategies is triggered by how individuals appraise certain situations (Lazarus and Folkman, 1984). Primary appraisal is related to individuals’ immediate evaluation of an event while secondary appraisal is individuals’ reflection on whether they have enough resources to deal with the situation. The appraisals will then activate individuals’ choice of emotion regulation strategies. According to Gross (1998, 2008), the regulatory strategies form a process model, there are antecedent-focused regulation and response-focused regulation. Antecedent-focused regulation efforts involve situation selection and situation modification to avoid contact with stressful events or altering the situation in the first place as preventive strategies (Gross and John, 2003). After the occurrence of an event, individuals may choose attentional deployment or cognitive change strategies to distract their attention from the stressful situation or modify their way of thinking and reappraise the situation. When individuals experience certain emotions, they are then able to engage in subsequent regulatory behaviors, such as suppressing emotions or faking unfelt emotions (Mikulincer and Florian, 1998).

One of the central themes of attachment theory relates to how people survive and cope with negative events and emotions (Bowlby, 1981). People seek a source of comfort from home, usually from their significant others. This behavior of comfort-seeking eventually turns into coping mechanisms when people leave home or interact with the wider society. Individuals with different attachment styles display various levels of distress-managing competence (Mikulincer and Shaver, 2016). Individual differences in attachment orientation are closely linked to individuals’ emotion regulation capabilities (e.g., Allen and Miga, 2010; Richards and Hackett, 2012; Mikulincer and Shaver, 2019). Secure individuals tend to be constructive, they are more likely to adopt an antecedent-focused strategy, to proactively deal with a situation which may provoke negative affect or reappraising an event by applying positive thinking (Cassidy, 1994). These constructive behaviors are a result of their positive relationship history with significant others. Secure individuals receive consistent support and attention from past interactions with caregivers, which enables them to be reassured when facing stressful situations and hold a positive view of self and others. When actually experiencing an emotion, secure people tend to be open to the feeling instead of deliberately denying or suppressing it (Cassidy, 1994). They are able to acknowledge the feeling and express it to significant others in the hope of sustaining or improving a relationship. Empirical evidence suggests that, compared with insecure individuals, secure people were more confident in their ability to cope with negative moods (Creasey et al., 1999).

For anxiously attached individuals, their relationship history is marked by caregivers’ inconsistency and unpredictability. Their sense of insecurity and fear of losing significant others hyperactivates their need to seek caregiver’s attention (Cassidy, 1994). Their strategies to regulate negative emotions are usually linked with intensifying these feelings to be reassured that significant others will not neglect or abandon them. This is supported in a lab experiment which shows that anxious individuals tended to self-report a higher level of distress, yet this was not detected in physiological measures, which suggested an exaggeration of distress (Maunder et al., 2006). By intensifying their feeling of vulnerability and helplessness, anxious individuals expect to capture significant others’ attention, whereas if they display sufficient competence in dealing with difficult situations, they may lose help.

Compared with anxious individuals’ intensification of feelings, avoidant individuals tend to suppress their negative feelings and choose to deal with these emotions alone without seeking others’ help or comfort (Main and Weston, 1982). The close relationship history is painful for avoidant individuals, it is usually characterized by unreliability and disappointment because of the unavailability of significant others. Thus, avoidant individuals distrust others and are overly self-reliant. They adopt a defensive approach when regulating emotions such as anxiety or distress, as they are unwilling to activate their attachment styles to recall past experiences (Main and Weston, 1982). Thus, they tend to choose emotion suppression or inhibition to block or reduce the chance of having to deal with close relationships. For avoidant individuals, seeking attention or help from significant others is often risky and may result in disappointment, thus, they are also less likely to seek support from others (Mikulincer and Shaver, 2007).

In the workplace, Affective Events Theory (Weiss and Cropanzano, 1996) suggest that events that happen at work trigger emotional responses, and these affective experiences influence people’s attitude and behavior at work. The emotion regulation capabilities derived from individuals’ attachment styles deeply influence how people perceive these events and subsequently influence their work outcomes. The direct effect of insecure attachment on affect and job satisfaction is supported by Kafetsios et al. (2014). They found that leader and follower insecure attachment styles were related to negative affect and lower job satisfaction. Also, consistent with the contagious effect of emotions (Barsade, 2002), leaders’ attachment insecurities were negatively related to follower positive affect and job satisfaction. In terms of emotion regulation strategies, research shows that insecure individuals engage in less adaptive coping strategies and are less likely to adopt problem-focused coping strategies (Johnstone and Feeney, 2015), which is not effective in terms of dealing with work stressors and are likely to result in poor physical and mental wellbeing (Regehr et al., 2013). This is consistent with theoretical assumptions, as anxious individuals are preoccupied with their emotions whereas avoidant individuals make efforts on blocking emotions, both focusing on dealing with emotions rather than solving problems. In leader-follower dyads, the interaction between attachment orientation and emotion regulation were found to be related to leader-follower relationships (Richards and Hackett, 2012). In particular, anxious individuals benefit from using emotion regulation strategies of suppression and reappraisal, which enable them to re-evaluate their emotions and the situations, possibly engaging in more constructive behaviors.

*Proposition 2. Employee attachment styles impact work outcomes by modulating emotional responses to workplace stressors.*

### Attachment and behavioral regulation

Attachment theory provides a comprehensive understanding of how individuals’ exploration and goal-pursuit behaviors are regulated, beginning in childhood and extending into adulthood. The concept of a “secure base” introduced by Bowlby (1973) emphasizes the role of significant others in providing a sense of safety that allows individuals to engage confidently with new environments. Ainsworth et al.’s (1978) “strange situation” study demonstrated this dynamic, showing that securely attached infants, assured of their caregiver’s presence, freely explored their surroundings, while anxious infants avoided exploration in favor of clinging to their caregivers, and avoidant infants disengaged emotionally, directing their attention to objects without genuine exploration. These early individual differences in attachment styles have significant implications across the lifespan, influencing how adults approach challenges and pursue goals.

Secure attachment encourages an openness to new experiences (Elliot and Reis, 2003) and a proactive approach to problem-solving and professional exploration (Mikulincer, 1997), supported by a regulatory system that harmonizes attachment needs with workplace exploration to enhance career growth and goal achievement (Feeney and Van Vleet, 2010; Dong et al., 2023). In a work context, this can manifest as taking calculated risks, engaging in skill development, and actively seeking feedback, which are instrumental for professional development. Conversely, anxious attachment, marked by a fear of rejection and preoccupation with maintaining approval from supervisors and peers (Bowlby, 1969), can restrict exploration by leading employees to question their own competence and avoid stepping beyond routine tasks (Ainsworth et al., 1978). Avoidantly attached individuals, often characterized by deactivated attachment systems due to previous negative relational experiences, may shy away from collaborative tasks or goal-oriented projects to evade potential interpersonal distress (Green and Campbell, 2000). These attachment-based motivations align with goal-orientation theory (Dweck, 1999) and regulatory focus theory (Higgins, 1987): securely attached employees adopt a promotion focus and a learning orientation, while insecurely attached employees may gravitate toward prevention-focused behaviors. Empirical research further supports these workplace implications; for example, securely attached adults exhibit greater curiosity, resilience, and adaptability, qualities that foster constructive responses to workplace challenges and adaptive problem-solving (Lopez et al., 1997). Thus, secure attachment serves as a “launch pad” (Bowlby, 1969) for professional exploration, shaping employees’ ability to thrive within dynamic and complex organizational environments.

These evidence informs workplace literature, as work has been identified as a major form of exploration in adulthood (Hazan and Shaver, 1990). It involves dealing with new information and knowledge and is consistent with individuals’ goal orientation process. Individuals’ motivation and competence to effectively explore is related to their work attitudes as well as working abilities. In the context of the workplace, attachment styles influence how employees engage with new tasks, adapt to changing environments, and pursue goals. Different from childhood exploration, where a secure base provides comfort for physical exploration, workplace exploration involves navigating complex organizational tasks, learning new skills, and interacting with colleagues to achieve professional growth. Securely attached employees, who feel confident in their abilities and supported by their organizational environment, are more likely to seek out challenging assignments, display resilience, and pursue creative problem-solving (Hazan and Shaver, 1990; Mikulincer, 1997). This reflects their proactive approach to work and openness to new experiences, which are critical for effective job performance and career development.

In Hazan and Shaver’s (1990) study, they found that attachment security was related to more positive work attitudes, as securely attached individuals are more willing and feel more comfortable to engage in exploratory behaviors. This mirrors Ainsworth’s experiment on strange situations, securely attached infants are more likely to feel protected for them to explore the surroundings. They have a positive self-image and more prone to be confident about their self-worth (Ainsworth et al., 1978). In contrast, attachment insecurities point to the unwillingness to explore which tend to predict more negative attitudes and less motivation. This is supported by the study of Richards and Schat (2011), they found that attachment avoidance and anxiety were related to employee turnover intentions and less organizational citizenship behavior.

Behavioral regulation is also applicable to another area of work literature, which is employee creativity. The motivation to explore and learn new things facilitate individuals’ ability to work creatively (e.g., De Stobbeleir et al., 2011). Creativity involves generation and implementation of ideas (Černe et al., 2018). As attachment theory proposes, individuals would withdraw from exploration behaviors if they experience feelings of threat or fear, thus, creative behavior is encouraged by individuals’ felt safety to explore. This is evidenced by a recent empirical study (Kirrane et al., 2019), which explored employee attachment styles and creativity. Results showed that insecure attachment negatively predicted employee creativity and this relationship was mediated by workplace relationships (relationship with leaders and the team), suggesting that successful workplace relationships provide employees with felt security to effectively explore.

Having described four regulatory mechanisms that may mediate the effects of attachment styles on work outcomes, we turn to the attachment activation processes as boundary conditions that may moderate the effects of attachment styles on work outcomes and also help to explain the inconsistent findings in the extant literature (Table 2).

**Table 2:** Summary of attachment styles and regulatory processes.

	Attachment security	Attachment anxiety	Attachment avoidance
Cognition regulation	Secure individuals process social information realistically and without bias, leading to less biased, more realistic interpretations of self and others. Work Implication: they are likely to make balanced decisions, have realistic self-assessments, and effectively interpret social cues, and are more likely to be effective in leadership roles.	Avoidant individuals show distorted cognition, focusing on potential rejection and abandonment, leading to negative attributions and excessive rumination on relationship threats. Work Implication: this can lead to overreacting to feedback, misinterpreting colleagues’ intentions, and reduced focus due to excessive worry.	Avoidant individuals tend to suppress attachment-related thoughts, leading to cognitive distancing and a reluctance to reflect on attachment experiences or process social information involving closeness. Work implication: they may struggle with collaboration and miss social cues, reducing their effectiveness in team settings.
Emotion regulation	Secure individuals maintain balanced emotional responses, effectively managing stress and showing resilience during challenges. Work implication: they are likely to remain calm under pressure, provide emotional support to others, and create a positive work atmosphere.	Anxiously attached individuals experience heightened emotional responses, such as anxiety and fear of rejection, often leading to emotional dysregulation and intense negative emotions in social situations. Work implication: they may frequently seek reassurance, creating additional emotional burdens on colleagues and disrupting workflow.	Avoidant individuals often suppress emotions, leading to emotional detachment. They may struggle with accessing or expressing feelings, particularly those related to vulnerability or intimacy. Work implication: their emotional withdrawal can create misunderstandings and limit effective communication in the workplace.
Behavioral regulation	Securely attached individuals feel confident to explore new environments due to the availability of a secure base and safe haven, leading to adaptive behaviors like problem-solving and proactive engagement in work tasks. Work implication: they tend to be more creative, take initiative, and adapt well to new challenges, enhancing their job performance.	Anxiously attached individuals are often hesitant to explore new environments, focusing instead on maintaining proximity to attachment figures. This avoidance of exploration can limit their learning and skill development. Work Implication: they may struggle with independent tasks, fear failure, and avoid taking risks, which hampers growth.	Avoidant individuals often withdraw from exploration due to painful past experiences, leading to a lack of engagement with new tasks or environments. Work implication: they may resist collaboration, show limited curiosity, and miss opportunities for personal and professional growth, impacting overall productivity and innovation.

*Proposition 3. Employee attachment styles influence work outcomes through regulating their behaviors in the workplace.*

## Attachment activation in the workplace

Personality is one of major causes and determinants of behavioral variance among people. In personality theorizing, one topic of long-standing debate is around how personalities function, whether they are stable, consistent across all situations or subject to change, specific to situations. For example, scholars have tried to understand the reasons for inconsistent performance of interview candidates across different situations (different interview exercises) (e.g., Lievens et al., 2006). Some of these arguments are reconciled by an interactionist perspective which takes into account both trait and situational approaches and stresses the importance of person and situation interaction. In an interactionist view, personality is defined as ‘intraindividual consistencies and interindividual uniqueness in propensities to behave in identifiable ways in light of situational demands’ (Tett and Guterman, 2000, p. 398). This definition acknowledges the relative stability of individual traits, at the same time, it underscores the importance of contextual stimuli that activate personality.

In a sense, individuals’ attachment styles capture such intrapersonal consistency and interpersonal uniqueness, as it involves individuals’ perceptions toward relationships and how individuals differ in terms of approaching relationships. However, a few conceptualization issues arise. First is whether to view individuals’ attachment styles as a personality or a relationship construct (Kobak, 1994). To view it as a personality is a theoretically straightforward way to categorize individual differences as the attachment system does trigger individual behavioral differences; however, the underlying concept is much more complex. When Hazan and Shaver (1987) first started to sketch the attachment process in romantic relationships, they discarded the categorization approach which separates individuals into three or four styles. Rather, they view it as a relational process accounting for the influence of the dyadic partner and the development of relationships. In the work literature, operationalizing it as personality allows researchers to empirically measure attachment styles and model the difference among employees. However, as the application of attachment theory in the management field starts to grow, the oversimplified conceptualization of the attachment style as a static trait impedes its development.

Second, attachment styles have largely been treated as a trait-like construct, focusing on stability rather than change; however, this also causes conceptual confusions (Davila et al., 1997). Global traits of individuals’ attachment styles are relatively stable, as Bowlby posits, they persist and are influential to an individual from ‘the cradle to the grave’. However, Bowlby also posits that attachment styles are sensitive to contexts and are able to accommodate for the intake of new information and new experiences that could potentially influence individuals’ perceptions toward an attachment relationship (Bowlby, 1973). A number of research studies have endorsed the variation in individuals’ behaviors under different contexts. For example, Collins et al. (2006) found that attachment-anxious individuals were more likely to hold pessimistic attributions to partner behaviors, but involvement in a high-quality relationship was able to alleviate the effect. Research also identified change in anxiously attached individuals from their global attachment tendencies when exposed to supportive environments (Pierce and Lydon, 2001). This is perhaps the reason why longitudinal investigations of attachment styles yield mixed results (Fraley and Roisman, 2019). Initial evidence was documented in early investigations which suggests that attachment styles formed in early life tend to retain and influence later relationships (see Fraley, 2002). However, more recent examinations suggest that modifications of chronic attachment styles are possible (Mikulincer and Shaver, 2007; Arriaga et al., 2018), individuals either become more insecure as a result of continuous exposure to helpless and stressful situations or experiencing major changes (Simpson et al., 2003; Arriaga et al., 2018), or enhance attachment security when engaged in high-quality close relationships at later stages in life (Carnelley and Rowe, 2007).

Based on the two conceptual issues, we propose that the modeling of attachment theory in the workplace should account for the relational process as well as contextual factors. we first draw from trait activation theory to capture the interaction between person and environment. Trait activation theory posits that individuals’ traits are likely to operate more strongly if the situational cues are trait-relevant (Tett and Burnett, 2003). Judge and Zapata (2015) argue that when comparing traits as resources, then one is expected to have better performance if an individual’s resources exceed situational demands. This line of argument applies to how individuals approach specific situations and exhibit different behaviors, and is particularly useful in denoting the trait-performance relationship, uncovering different contextual factors that could potentially weaken or strengthen the relationship.

The application of trait activation theory in the context of attachment styles emphasizes that specific workplace cues can activate attachment-related responses, depending on the alignment between these cues and attachment needs. For attachment styles to be meaningfully triggered, the workplace context must contain stimuli relevant to an individual’s attachment system—contexts that are perceived as relationally challenging, high-stakes, or otherwise demand high levels of interpersonal interaction often fit these criteria. For instance, high-stakes environments, such as project deadlines or performance evaluations, can amplify stress and activate attachment insecurities, particularly for anxiously attached individuals who seek reassurance and validation. In these high-stress situations, their self-regulatory capacities may become strained, leading to heightened anxiety and possibly disruptive behaviors if reassurance is lacking.

For the context to be attachment-relevant, it has to activate and provoke the attachment system. For example, individuals develop working models of attachment anxiety as a result of lacking consistent support and attention. In other words, their attachment anxiety is most likely to be triggered and influence their behavior when facing threats without help from significant others. They are short of the resources needed to deal with stressful and challenging situations. Thus, there might be a relationship between attachment anxiety and negative and disruptive behavior. However, when given enough attention and guidance, the strength of this relationship will be weakened. Individuals who develop attachment avoidance perceive themselves as independent and have little desire to build close relationships, they are used to relying on themselves, thus they have enough resources in terms of independence, and when given more autonomy, they are more likely to exhibit more positive work-related behaviors. We elaborate on the contextual factors in the workplace below.

We propose that work-related trait activation cues serve as contextual factors that moderate the relationship between attachment styles, regulatory process and relevant work outcomes. For attachment-related situations to be relevant, we selected work contexts that would trigger individual attachment system functioning. It should be noted that the specific work contexts we provided below not an exhaustive list but ones that are relevant to individual attachment-related traits. First, we take task-level requirement as an example and elaborate on the situation of creativity requirement. As we detailed before in sections on behavioral regulation, secure individuals are more willing to explore, and are likely to exhibit creative skills. Thus, creativity-oriented task may trigger secure individuals’ comfort with exploration and innovation, activating behaviors aligned with openness and adaptability (Chen et al., 2023). This activation enables a secure attachment style to positively influence self-regulatory strategies, ultimately leading to enhanced creative performance. However, for insecure individuals, the same creativity task may evoke feelings of uncertainty or discomfort, leading to more defensive or avoidant regulatory responses, which negatively affect performance. Another common workplace scenario involves tasks that require varying levels of supervision versus flexibility. Tasks requiring high supervision may support anxious individuals by offering reassurance and external validation, thus positively moderating their regulatory capabilities, and enhancing performance. Conversely, avoidant individuals may experience supervision as a threat to autonomy, prompting stress or resistance, which undermines their regulatory effectiveness. In this case, the supervision cue moderates the mediation by reinforcing the anxious individual’s regulatory efficacy while diminishing that of avoidant individuals.

Collaborative tasks also serve as a boundary condition that distinctly activates attachment dynamics, as they require significant social engagement. Securely attached individuals may thrive in these settings, exhibiting open communication and trust. Conversely, avoidantly attached individuals, who prefer autonomy, may find such settings triggering, as they perceive enforced collaboration as a challenge to their independence, potentially resulting in withdrawal or reduced cooperative effort. Also, high levels of supervision and autonomy provide additional boundary conditions that influence attachment activation. Anxious individuals often perform better under close supervision, as it provides the external validation and support they crave. This environment aligns with their attachment needs, supporting self-regulation and enhancing task performance. In contrast, avoidantly attached individuals tend to excel in autonomous roles, where their preference for self-reliance can be fully expressed without perceived threats to their independence. Autonomy-supportive environments, therefore, reinforce positive work behaviors among avoidant individuals while minimizing the activation of attachment-related stress.

Another example would be social interaction cues in the workplace. These cues activate attachment-based interpersonal regulatory processes. Secure individuals are likely to respond to such cues with positive social behaviors, enhancing trust, communication, and empathy, which improves performance outcomes. Insecure individuals, however, may experience these cues as challenging, resulting in withdrawal or heightened stress, thereby undermining their regulatory capabilities in social interactions and negatively affecting performance. For secure individuals, social cues strengthen the regulation-to-outcome pathway by enabling effective interpersonal regulation, while for avoidant or anxious individuals, the same cues can disrupt the regulatory process, yielding poorer outcomes.

On a broader level, we postulate that organizational culture could serve as one of the most salient features for attachment-relevance (Yip et al., 2018). For example, supportive organizational culture, where high levels of perceived support are present, particularly benefits anxious individuals, as it aligns with their need for reassurance and proximity. This environment enables anxious individuals to regulate their emotions more effectively and engage more fully in their work, enhancing their performance (Wu and Parker, 2017). Securely attached employees also benefit from such support, though their need for validation is less pronounced, suggesting a weaker impact on their regulation and work outcomes compared to anxious employees. Avoidant individuals, however, experience improved outcomes in environments that emphasize autonomy rather than support, as autonomy-supportive leadership aligns with their preference for self-sufficiency and independence (Wu and Parker, 2017). In competitive organizational cultures, avoidant individuals are likely to excel due to their comfort with self-reliance and competitive focus, while anxious individuals may experience regulatory challenges, as their self-doubt can be exacerbated by the lack of cooperative support, hindering performance. Additionally, high-organizational justice environments have been shown to facilitate prosocial behaviors among securely attached individuals, as such settings satisfy their orientation toward fairness and stability (Syna Desivilya et al., 2006). Collectively, these organizational cues serve as critical moderators by either facilitating or inhibiting the self-regulatory processes needed to translate attachment orientations into effective work performance, depending on the alignment between the organizational environment and individual attachment-related needs (Yip et al., 2018) (Table 3).

**Table 3:** Summary of the activation process.

Attachment style	Trait activation cues	Expected work outcomes	Theoretical justification
Secure	Creativity-oriented tasks, social interaction cues, high-organizational justice	Positive outcomes: increased creativity, effective collaboration, enhanced prosocial behaviors. Negative outcomes: minimal; stable performance across contexts.	Creativity tasks activate secure individuals’ openness to exploration, facilitating creative output. Social cues trigger positive interpersonal regulation, enhancing teamwork, trust, and empathy. High organizational justice aligns with their value for fairness, promoting prosocial behaviors and stable performance in varied contexts.
Avoidant	Autonomy-supportive tasks, competitive organizational culture, social interaction cues	Positive outcomes: increased task productivity, improved goal-oriented performance. Negative outcomes: decreased collaboration, lower social engagement.	Autonomy-supportive tasks match avoidant individuals’ preference for independence, fostering task engagement. Competitive cultures align with their self-reliance, activating goal-focused behaviors. However, social cues may prompt withdrawal due to discomfort with interpersonal closeness, reducing collaborative and social performance.
Anxious	Supervised tasks, supportive organizational culture, competitive organizational culture	Positive outcomes: improved task performance under supervision, higher engagement in supportive settings. Negative outcomes: lower performance in competitive contexts, heightened stress.	Supervised tasks offer reassurance, aiding anxious individuals’ regulation and improving task outcomes. Supportive cultures engage them by fulfilling their need for validation. In contrast, competitive environments may trigger self-doubt and regulatory challenges, leading to reduced performance and increased stress.

## Theoretical and practical implications

Our study significantly contributes to the literature on attachment theory in the workplace by providing a comprehensive theoretical integration and advancement of the theory, drawing on extant theorization and empirical evidence in the domains of cognitions, relationships, emotions, and behaviors. This is the first paper, to our knowledge, that synthesizes these diverse areas into an overarching framework, elucidating the mechanisms through which the attachment system functions in the workplace. By doing so, we highlight how attachment styles influence cognitive processes, emotional regulation, and behavioral orientations in a professional context.

Attachment theory traditionally focuses on how individuals form and maintain relationships based on their early interactions with caregivers, which then influences their relational patterns throughout life. Our framework extends this foundational understanding into the workplace, a domain characterized by distinct social dynamics and goal-oriented activities. We propose that attachment styles shape how employees perceive workplace dynamics, manage their emotions, interact with colleagues, and engage in work-related behaviors. For instance, securely attached individuals are likely to have positive perceptions of their work environment, effectively manage workplace stress, build strong interpersonal relationships, and demonstrate proactive work behaviors. Conversely, those with insecure attachment styles may face challenges in these areas, influencing their overall work performance and job satisfaction. Traditional developmental models focus primarily on close personal relationships, such as those with caregivers or romantic partners. In contrast, our framework addresses how attachment styles manifest in a wider array of workplace interactions, including relationships with colleagues, supervisors, and teams.

Additionally, our framework employs trait activation theory to identify the boundary conditions under which attachment styles operate differently. Trait activation theory posits that certain situational cues can activate specific traits, thereby influencing behavior. By integrating this theory, we offer a nuanced explanation for the variability in how attachment styles manifest in different workplace contexts. For example, a highly collaborative work environment may amplify the attachment-related needs for connection in anxiously attached individuals, while a highly autonomous role may accentuate the self-reliance tendencies in avoidantly attached individuals. This approach not only provides a deeper understanding of the attachment mechanisms at play but also addresses the inconsistent findings in previous empirical research by highlighting the contextual factors that moderate these effects.

Furthermore, our theoretical integration underscores the dynamic interplay between cognitive, emotional, and behavioral regulation in the workplace. We elucidate how attachment styles influence employees’ cognitive appraisals of work situations, their emotional responses to workplace challenges, and their resultant behaviors. By offering a multi-dimensional perspective, our framework captures the complexity of attachment processes in professional settings, providing a richer and more comprehensive understanding of how attachment theory can inform organizational behavior.

This framework informs managerial practice of the importance of employee individual differences in terms of their attachment styles. First, managers need to pay attention to these individual differences and offer help to employees when necessary. Organizations could provide training regarding how managers could effectively initiate communications and sustain relationships with employees. Also, job design could act as an important trait-relevant activation factor (Yip et al., 2018). More interdependent tasks that require collaborations could be offered to anxiously-attached employees. For avoidantly attached individuals, they could benefit from roles that require more autonomy.

## Limitations and future research directions

Despite its contributions, the theoretical framework we propose has limitations in its scope. It primarily focuses on attachment styles as regulatory devices, emphasizing the regulatory processes. However, other potential mechanisms may mediate the relationship between attachment styles and work outcomes. For instance, workplace emotions, such as positive and negative affect, could serve as potential mediators. According to Richards and Schat (2011), affective experiences significantly impact employees’ motivation, decision-making, and interpersonal interactions. Understanding how these emotional states mediate the influence of attachment styles can provide deeper insights into employee behavior and performance. For example, securely attached individuals might experience more positive emotions, leading to higher engagement and productivity, whereas insecurely attached individuals might be more susceptible to negative emotions, impacting their job satisfaction and effectiveness.

While our research highlights situational factors as potential moderators, future research could delve deeper into the intricate relationship between attachment styles and workplace dynamics by examining additional moderators and individual differences that shape these interactions. Our research emphasizes the role of situational factors, such as task characteristics and organizational culture, as potential moderators of attachment-driven behaviors. However, future studies should extend this line of inquiry by exploring how individual resources, such as political skills and interpersonal skills, may moderate these relationships. Political skills, which encompass the ability to navigate complex organizational dynamics and influence others effectively, could buffer the negative effects of insecure attachment styles by enhancing individuals’ adaptability and strategic interaction with colleagues and leaders. Similarly, interpersonal skills, including communication proficiency and conflict resolution capabilities, could mitigate the adverse impacts of attachment insecurities by improving relationship quality and promoting team cohesion. Investigating these individual differences as moderators would not only enrich our understanding of attachment theory in the workplace but also facilitate the development of tailored interventions aimed at supporting employees with varying attachment styles.

Furthermore, the attachment system, reflecting an individual’s socio-personality and relational orientation, functions as a regulatory mechanism that governs perceptions, motivations, and behaviors within social and organizational contexts. This framework offers a valuable lens for examining the “dark side” of workplace behavior, particularly in leadership. Dysfunctional personality traits associated with insecure attachment, such as the propensity toward narcissism among avoidant individuals, suggest that attachment theory can help explain maladaptive leadership behaviors, including narcissistic leadership and abusive supervision (Pistole, 1995). Future research could investigate the specific pathways through which leaders’ insecure attachment styles manifest in destructive leadership behaviors, such as controlling, dismissive, or punitive actions that undermine team morale and performance. Understanding these dynamics could significantly contribute to leadership development by highlighting the importance of addressing attachment insecurities in leaders, thereby mitigating the detrimental effects on their teams. For instance, interventions focusing on self-awareness and relational management could be particularly beneficial for leaders prone to avoidant or anxious attachment styles, enhancing their ability to foster supportive and effective team environments.

Additionally, an important avenue for future research involves examining the formation and evolution of attachment relationships in organizational settings, particularly during critical periods such as onboarding and organizational socialization. While attachment styles are often conceptualized as stable, trait-like dispositions in management studies, psychological research indicates that attachment orientations can be dynamic and subject to change through new relational experiences (Mikulincer and Shaver, 2016; Arriaga et al., 2018). Exploring how new employees develop attachment relationships with their leaders and peers in the early stages of their employment could provide valuable insights into the mechanisms of secure attachment formation and its impact on job satisfaction, engagement, and performance. Investigating factors that facilitate the development of secure attachment in the workplace, such as leader behaviors, team climate, and organizational support systems, could inform the design of onboarding and integration programs that promote positive relational dynamics from the outset. Moreover, longitudinal studies examining the evolution of these relationships over time would shed light on how organizational practices and policies can either reinforce or reshape employees’ attachment orientations, with implications for long-term employee well-being and retention.

Another promising direction for future research is the investigation of attachment styles in technologically mediated work environments, particularly in the context of remote work, digital communication, and artificial intelligence (Gillath et al., 2021; Tang et al., 2023; Kim et al., 2022). As work becomes increasingly virtual, the traditional mechanisms through which attachment styles influence workplace interactions may be altered. For instance, the absence of physical presence and in-person social cues in virtual settings may exacerbate feelings of isolation and neglect among anxiously attached individuals, while offering avoidant individuals a perceived increase in autonomy (Benoit and DiTommaso, 2020). Future studies should explore how attachment styles are activated and expressed in digital workspaces, and how these environments can be structured to support diverse attachment needs. Integrating attachment theory with other psychological constructs, such as resilience and emotional intelligence, could provide a more comprehensive framework for understanding how individual differences affect adaptation to technological change. Such research would not only advance theoretical knowledge but also guide practical strategies for managing attachment-related challenges in evolving work environments.

On the practical side, managers can improve employee outcomes by tailoring their approaches to fit the attachment styles of individual employees. For example, providing task autonomy and clear expectations may be particularly beneficial for avoidantly attached individuals, who tend to value independence and prefer structured, predictable environments. Specific task assignments that allow for solitary, self-paced work—such as individual projects with minimal supervision—could support these employees’ needs, helping them feel secure and reducing potential stress. Conversely, anxiously attached individuals might benefit more from team-based tasks that offer regular feedback and reassurance, fostering a sense of connectedness and security. Managers can play an active role in creating a supportive environment by recognizing these varying needs and adjusting task assignments accordingly.

Organizations can apply this framework to design interventions that foster secure attachment orientations and positive relational dynamics from the outset. For example, during onboarding, team-building exercises and structured mentorship programs can help new employees develop supportive relationships with leaders and peers. Training programs that promote empathy, communication, and self-awareness may enhance leaders’ ability to engage with employees’ diverse attachment needs, fostering a more inclusive and supportive workplace. Additionally, workshops focused on relationship management and emotional intelligence can provide employees with tools to navigate attachment-related challenges, ultimately improving team cohesion and reducing potential conflicts.

By addressing these limitations and exploring these future research directions, we can further our understanding of the complex interplay between attachment styles and workplace dynamics, ultimately contributing to the development of more supportive and effective organizational environments.

## Conclusion

This paper advances the understanding of attachment theory within workplace settings by providing a conceptual framework that elucidates how attachment styles shape work outcomes through distinct regulatory mechanisms. By integrating insights from broader attachment research with organizational behavior literature, we offer a novel perspective on the activation and regulation processes that influence employee behaviors. This framework not only addresses existing inconsistencies in empirical findings but also serves as a foundation for future research to explore targeted interventions aimed at enhancing employee well-being and organizational effectiveness. Ultimately, our work underscores the significance of considering attachment dynamics as a key factor in understanding the psychological drivers of workplace behavior.
